# Chromosomal organization of the ribosomal RNA genes in the genus *Chironomus* (Diptera, Chironomidae)

**DOI:** 10.3897/CompCytogen.v9i2.9055

**Published:** 2015-05-22

**Authors:** Larisa Gunderina, Veronika Golygina, Andrey Broshkov

**Affiliations:** 1 Institute of Cytology and Genetics SB RAS, Academician Lavrentiev avenue 10, Novosibirsk, 630090, Russia; 2 Novosibirsk State University, Pirogova str. 2, Novosibirsk, 630090, Russia

**Keywords:** *Chironomus*, 5.8S rDNA, ribosomal gene localization, polytene chromosomes, NOR, gene mapping, FISH

## Abstract

Chromosomal localization of ribosomal RNA coding genes has been studied by using FISH (fluorescence *in situ* hybridization) in 21 species from the genus *Chironomus* Meigen, 1803. Analysis of the data has shown intra- and interspecific variation in number and location of 5.8S rDNA hybridization sites in 17 species from the subgenus *Chironomus* and 4 species from the subgenus *Camptochironomus* Kieffer, 1914. In the majority of studied species the location of rDNA sites coincided with the sites where active NORs (nucleolus organizer regions) were found. The number of hybridization sites in karyotypes of studied chironomids varied from 1 to 6. More than half of the species possessed only one NOR (12 out of 21). Two rDNA hybridization sites were found in karyotypes of five species, three – in two species, and five and six sites – in one species each. NORs were found in all chromosomal arms of species from the subgenus *Chironomus* with one of them always located on arm G. On the other hand, no hybridization sites were found on arm G in four studied species from the subgenus *Camptochironomus*. Two species from the subgenus *Chironomus* – *Chironomus balatonicus* Devai, Wuelker & Scholl, 1983 and Chironomus “annularius” sensu Strenzke, 1959 – showed intraspecific variability in the number of hybridization signals. Possible mechanisms of origin of variability in number and location of rRNA genes in the karyotypes of species from the genus *Chironomus* are discussed.

## Introduction

The ribosomal RNA genes in eukaryotic genomes are multiply repeated and form the family of ribosomal genes. They are arranged in clusters comprising hundreds of tandemly repeated units, each consisting of three genes – 18S, 5.8S, and 28S rRNA – separated by transcribed and untranscribed intergenic spacers (Long and David 1980). The clusters of ribosomal RNA genes in chromosomes are located to the nucleolus organizer regions (NORs). Two methods are currently used to detect these regions, namely, FISH (fluorescence *in situ* hybridization) with rDNA probes, allowing for localization of rRNA genes, and silver nitrate staining, allowing for detecting their activity. Studies involving numerous animal and plant groups have demonstrated that the number of NORs and their location on chromosomes may differ not only in distant species, but also in closely related ones. Research into NOR variation in karyotypes has clarified the patterns in chromosome evolution of many insect groups (Cabrero and Camacho 2008, Cabral-de-Mello et al. 2010, 2011, Grzywacz et al. 2011, Oliveira et al. 2011, Neto et al. 2013).

The chromosome evolution of the species belonging to the genus *Chironomus* Meigen, 1803 has been studied in much more detail as compared with the other insect groups owing to the presence of polytene chromosomes with a distinct species-specific banding pattern in the nuclei of their salivary glands (Keyl 1962, Martin 1979, Wuelker 1980, Shobanov 2002, Kiknadze et al. 2008). Seven arms of the *Chironomus* haploid chromosome set comprise over 1000 robustly identifiable bands (chromosome markers), and the homology of banding sequences in chromosomes of different species can be detected using the mapping system devised for this genus (Keyl 1962). Correspondingly, comparison of the banding sequences reliably detects the changes in the linear structure emerging in chromosomes (inversions, deletions, duplications, and translocations). A high density of the known markers – chromosome bands – in chironomid chromosomes makes it possible to find even small chromosome rearrangements involving only one or two bands (Kiknadze et al. 2004a, b). Studies of intraspecific and interspecific polymorphism in banding sequences of the chironomid polytene chromosomes have provided the insight into emergence and spreading patterns of chromosome polymorphism in the distribution ranges of individual species. They also allowed to better understand the phylogenetic relationships between species as well as to reconstruct cytogenetic evolution of the genus *Chironomus* (Keyl 1962, Martin 1979, Shobanov 2002, Kiknadze et al. 2004a, b, 2008).

On the other hand, the information about the number and location of NORs in the chromosomes of *Chironomus* species is mainly based on a phase contrast analyses of acetorcein-stained chromosomes (Beermann 1960). Silver nitrate staining (Lentzios and Stocker 1979) and *in situ* hybridization (Hollenberg 1976, Eigenbrod 1978, Razmakhnin et al. 1982) have been used to study NORs of only a few *Chironomus* species. The absence of these data prevents clarification of the patterns for chromosome evolution of the rRNA gene family in the genus *Chironomus*.

The goal of this work was to study the chromosomal localization of the rRNA locus in the genus *Chironomus* species by means of FISH. The DNA sequences of chironomid species from the rRNA locus carrying 5.8S rRNA gene (5.8S rDNA) and the internal transcribed spacer (ITS-1) separating 18S and 5.8S rRNA genes were selected as the probes.

## Material and methods

The IV instar larvae of 21 *Chironomus* species belonging to the subgenera *Chironomus* and *Camptochironomus* Kieffer, 1914 sampled in aquatic bodies of the Novosibirsk region, Russia, were examined. The larvae of North-American species *Camptochironomus dilutus* Shobanov, Kiknadze & Butler, 1999 were obtained from the laboratory culture maintained at the Institute of Biology of Inland Waters, Russian Academy of Sciences (Borok, Yaroslavl region, Russia). Seven examined species of the subgenus *Chironomus* belong to the group of *Chironomus plumosus* sibling species, namely, *Chironomus agilis* Schobanov & Djomin, 1988, *Chironomus balatonicus* Devai, Wuelker & Scholl, 1983, *Chironomus borokensis* Kerkis, Filippova, Shobanov, Gunderina & Kiknadze, 1988, *Chironomus entis* Schobanov, 1989, *Chironomus muratensis* Ryser, Scholl & Wuelker, 1983, *Chironomus nudiventris* Ryser, Scholl & Wuelker, 1983, and *Chironomus plumosus* (Linnaeus), 1758. These species as well as Chironomus “annularius” sensu Strenzke, 1959, *Chironomus riparius* Meigen, 1804, *Chironomus cingulatus* Meigen, 1830, *Chironomus nuditarsis* Keyl, 1961, and *Chironomus sororius* Wuelker, 1973 belong to the «thummi» cytocomplex, characteristic of which is the arm combination AB CD EF G in the chromosomes of their karyotype. *Chironomus dorsalis* Meigen, 1818, *Chironomus luridus* Strenzke, 1959, *Chironomus melanescens* Keyl, 1961, and *Chironomus pseudothummi* Strenzke, 1959 (arm combination, AE BF CD G) belong to the pseudothummi cytocomplex and *Chironomus lacunarius* Wuelker, 1973 (arm combination, AD BC EF G), to the lacunarius cytocomplex. The four species of the *Camptochironomus* subgenus – *Camptochironomus dilutus*, *Camptochironomus pallidivittatus* sensu Beermann, 1955, *Camptochironomus setivalva* Shilova, 1957, and *Chironomus tentans* Fabricius, 1805 – belong to the camptochironomus cytocomplex (arm combination, AB CF ED G).

The larvae were fixed with 96% ethanol (for further DNA extraction) or 3: 1 *v/v* of 96% ethanol and glacial acetic acid (for making preparations of salivary gland polytene chromosomes for FISH hybridization) and stored at –20 °C. Species were identified according to morphological characteristics of larvae and by cytogenetic analysis of banding patterns of polytene chromosomes from salivary glands (Kiknadze et al. 1991).

Genomic DNA was isolated from individual larvae using a DNeasy Blood and Tissue Kit (QIAGEN) according to the manufacturer’s protocol. DNA probes were produced by polymerase chain reaction (PCR) with the primers 5’-GTAACAAGGTTTCCGTAGG-3’ (chir5F) and 5’-CGACACTCAACCATATGTACC-3’ (chir5R) (Gunderina and Katokhin 2011, Gunderina 2014). Either genomic DNA or isolated, purified, and characterized DNA fragments with a length of ~480 bp from the 18S–5.8S rDNA region of the chironomid species listed in Table 1 were used as a template. The rDNA probes were labeled with biotin-11-dUTP or digoxigenin-11-dUTP (Roche, Germany). The DNA probes were precipitated according to a standard technique with fragmented salmon DNA as a DNA carrier. The ITS-1 and 5.8S rDNA sequences used as a DNA probes were aligned to characterize the interspecific differences using the MUSCLE program (Edgar 2004) (http://www.ebi.ac.uk). Molecular genetic analysis of these sequences was conducted using the MEGA 6 software package (Tamura et al. 2013). For NJ-tree construction sequences ITS1 and 5.8S rDNA of *Drosophila melanogaster* M21017 from GenBank database were used as an outgroup.

**Table 1.** The DNA probes used in the work.

	Species	DNA probe	GenBank accession number
1	*Chironomus agilis*	*ITS-1 + 5.8S_agi*	GU053584
2	Chironomus “annularius”	*ITS-1 + 5.8S_ann*	HQ656600
3	*Chironomus balatonicus*	*ITS-1 + 5.8S_bal*	GU053586
4	*Camptochironomus dilutus*	*ITS-1 + 5.8S_dil*	KP985232
5	*Chironomus dorsalis*	*ITS-1 + 5.8S_dor*	GU053590
6	*Chironomus muratensis*	*ITS-1 + 5.8S_mur*	GU053605
7	*Camptochironomus pallidivittatus*	*ITS-1 + 5.8S_pal*	KP985231
8	*Chironomus plumosus*	*ITS-1 + 5.8S_plu*	GU053597
9	*Chironomus riparius*	*ITS-1 + 5.8S_rip*	GU053603
10	*Camptochironomus setivalva*	*ITS-1 + 5.8S_set*	-
11	*Chironomus tentans*	*ITS-1 + 5.8S_ten*	KP985230

For FISH, the polytene chromosomes were prepared from the larvae fixed with 3 : 1 *v/v* of 96% ethanol and glacial acetic acid according to the following procedure. A larva was placed into 70% ethanol to extract its salivary glands and transfer them onto a glass slide into a drop of 45% acetic acid. The cells were separated from secretion by removing it from the glass, gently covered with a cover glass, and squashed, removing excess acid with filter paper. The ready preparation was placed for 10–15 min onto a metal table cooled with liquid nitrogen to remove the cover glass; the slide was then kept for 5 min at a room temperature, 5 min in 70% ethanol, and air-dried for 1 week.

FISH was conducted according to the following protocol. The preparations were air-dried for one week. They were then incubated with RNase A (100 mg/ml in 2× SSC) for 1 h at 37 °C, washed at a room temperature for 5 min with 2× SSC, dehydrated with alcohols (70, 90, and 96% ethanol, 5 min in each), and air-dried for 10 min. Then the slide was incubated with 0.02% pepsin in 10 mM HCl for 6 min at 37 °C, washed with a series of phosphate buffers (5 min in PBS, 5 min in PBS with 50 mM MgCl_2_, 10 min in PBS with 50 mM MgCl_2_ and 1% formaldehyde, and again in PBS and PBS with 50 mM MgCl_2_, 5 min each) at a room temperature, and dehydrated in alcohols as described above. DNA probe (dissolved in 20 µl of 2× SSC with 50% deionized formamide for 1 h at 37 °C in a thermoshaker at 800 rpm) was applied to dry slide, covered with a cover glass, and incubated for 12–15 h at 37 °C in a humid chamber. The slides were then washed in a shaker (100 rpm, 37 °C) two times for 10 min in 2× SSC with 50% deionized formamide and 0.1% NP40, two times for 5 min in 2× SSC, two times for 5 min in 0.2× SSC, and one time in 4× SSC with 3% BSA; then antibody solution (20 µl) was added, the slide was covered with a cover glass and incubated in a humid chamber at 37 °C for 40 min. The DNA probes labeled with biotin or digoxigenin were detected using the antibodies labeled with the fluorochromes avidin-Alexa fluor®488 or Cy3, respectively. The antibodies were diluted with 4× SSC containing 3% BSA (1–2 µl antibodies per 100 µl reaction mixture) and dissolved for 1 h in a thermoshaker (800 rpm, 37 °C) in parallel with washings after the hybridization with DNA probes. On completion of the incubation with antibodies, the slides were washed in a shaker (110 rpm, 37 °C) three times, 5 min each, in 4× SSC with 0.1% NP40; dehydrated with alcohols; air-dried for 15 min; mounted in a DAPI-containing antifade; and covered with a cover glass. Homologous DNA probes (the karyotype and DNA probe belongs to the same species) and heterologous DNA probes (the karyotype and DNA probe belongs to different species) were used for FISH.

The slides were examined using the equipment of the Joint Access Center for Microscopy of Biological Objects with the Siberian Branch of the Russian Academy of Sciences, namely, AxioPlan2 Imaging microscope and Axio Cam HRc CCD camera with the help of Isis 4 software package (Zeiss, Germany).

Mapping of polytene chromosomes in arms A, C, D, E and F was done according to Keyl–Devai system (Keyl 1962, Devai et al. 1989). Arm B was mapped according to Keyl–Devai system (Keyl 1962, Devai et al. 1989) in *Chironomus riparius*, according to Maximova–Shobanov system (Maximova 1976, Shobanov 1994) in species of *Chironomus plumosus* group of sibling species, and was not mapped in other species studied in this paper. Arm G was mapped according to Keyl–Hägele system (Keyl 1957, Hägele 1970) in *Chironomus riparius* and according to Maximova–Shobanov system (Maximova 1976, Shobanov 1994) in *Chironomus plumosus*, *Chironomus borokensis* and *Chironomus balatonicus*. Mapping of polytene chromosomes of species from the subgenus *Camptochironomus* was done according to Beermann system (Beermann 1955).

## Results

**Figure 1.**
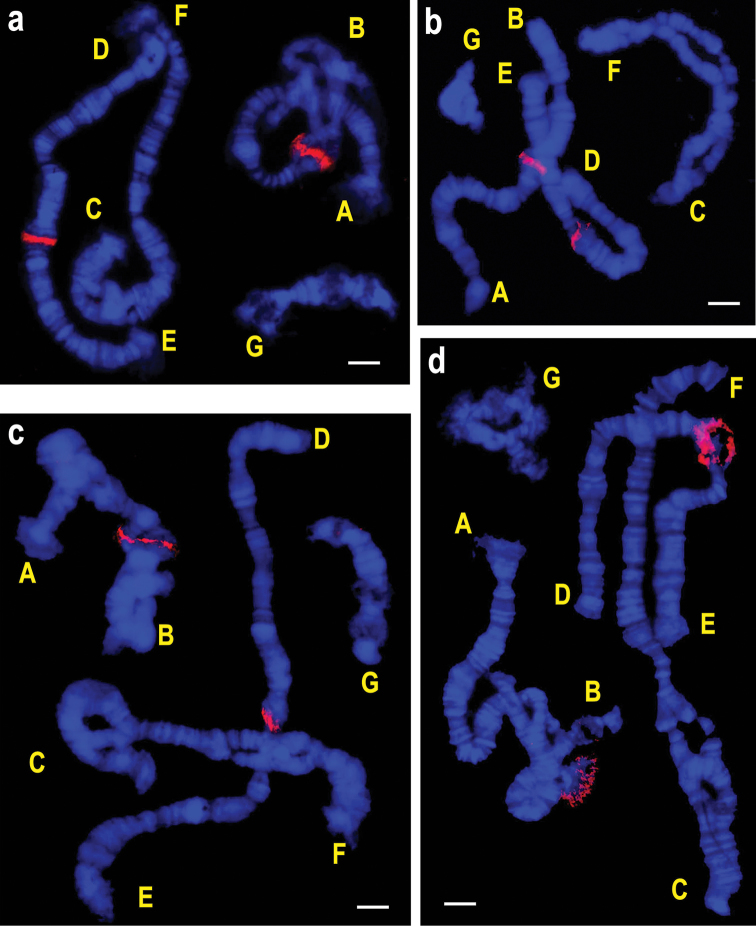
FISH of homologous (**a**) and heterologous (**b–d**) rDNA probes on the polytene chromosomes of *Chironomus tentans*. **a** *ITS-1 + 5.8S_ten* (Cy3) **b** *ITS-1 + 5.8S_pal* (Cy3) **c** *ITS-1 + 5.8S_dil* (Cy3) **d** *ITS-1 + 5.8S_set* (Cy3). Letters designate chromosomal arms. Bar = 10 µm.

**Figure 2.**
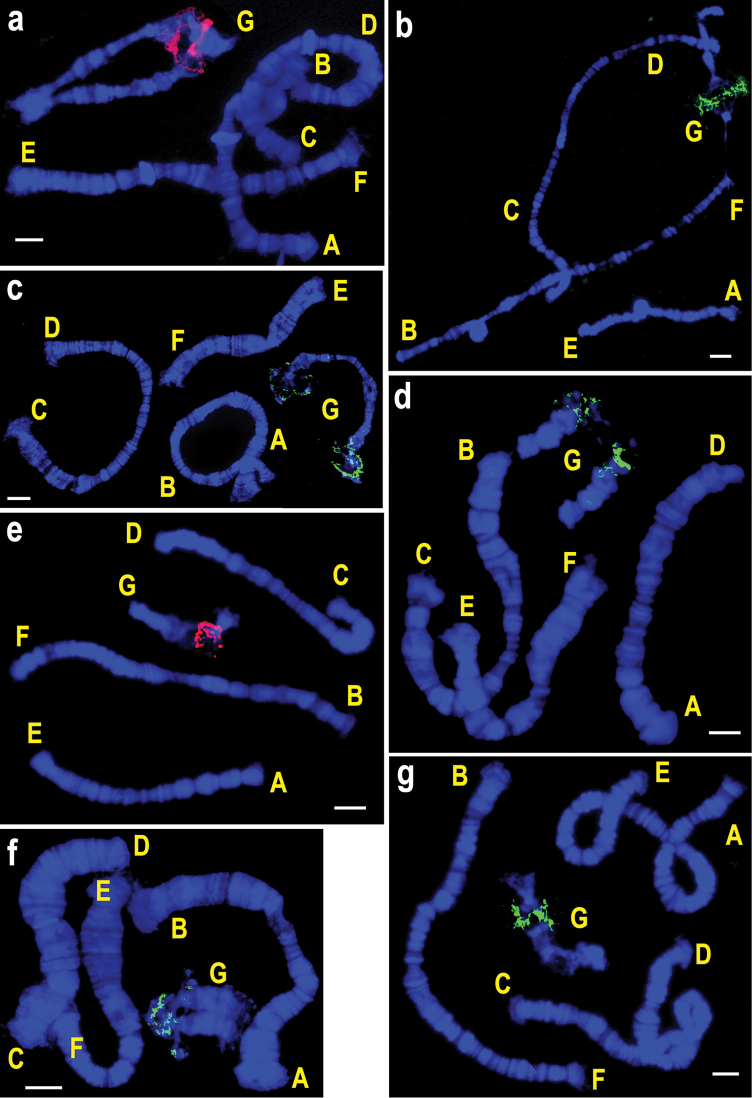

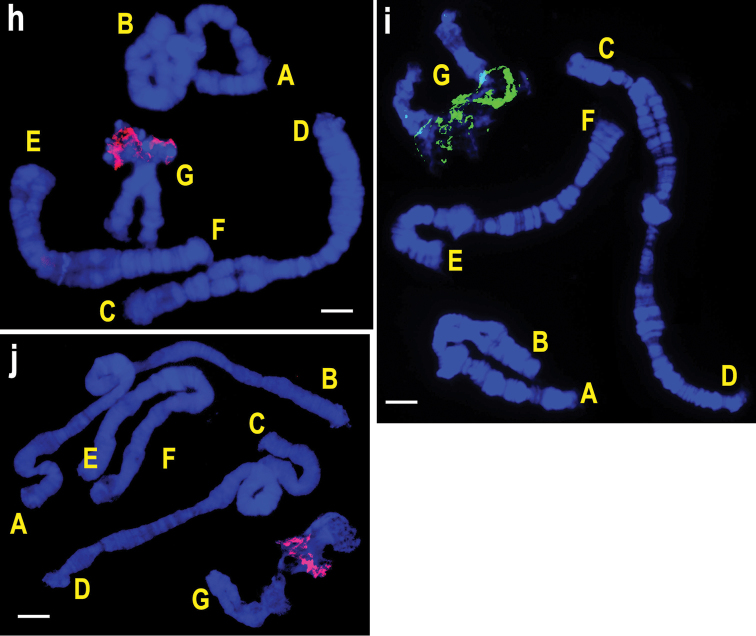
FISH of rDNA probes on polytene chromosomes of species from the subgenus *Chironomus* with one NOR in karyotype. **a** *Chironomus borokensis* **b** *Chironomus dorsalis* **c** *Chironomus entis* **d** *Chironomus lacunarius* **e** *Chironomus luridus* **f** *Chironomus nuditarsis* **g** *Chironomus melanescens* **h** *Chironomus plumosus* **i** *Chironomus sororius* **j** *Chironomus riparius*. Letters designate chromosomal arms. Bar = 10 µm.

**Figure 3.**
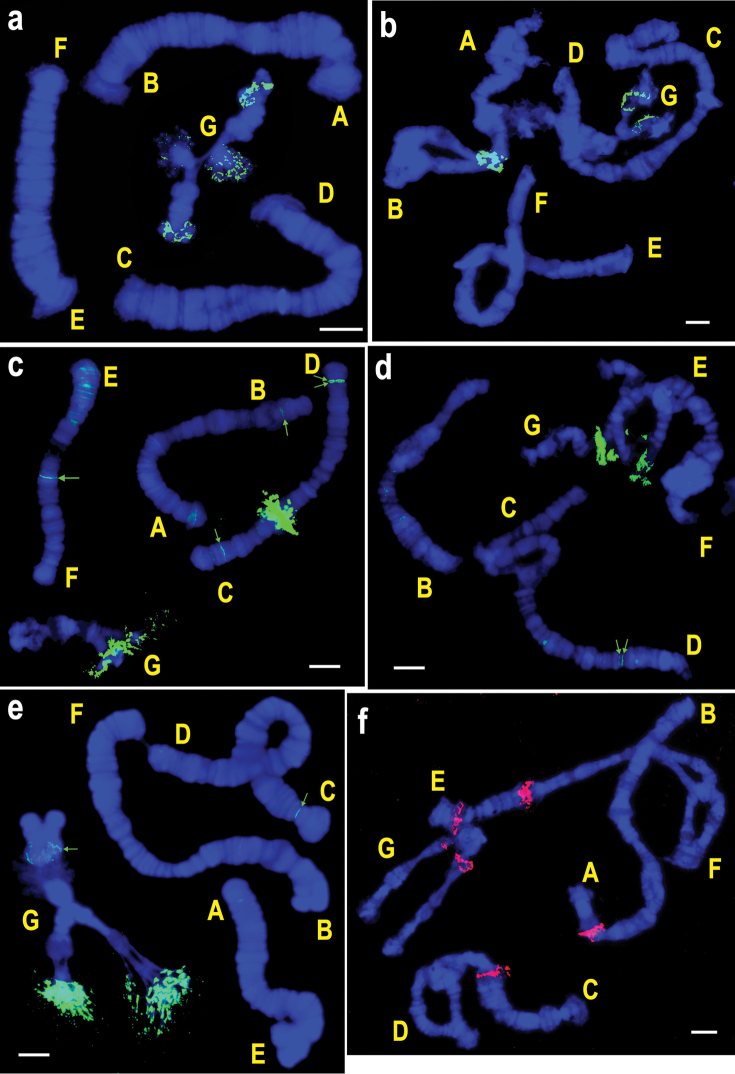

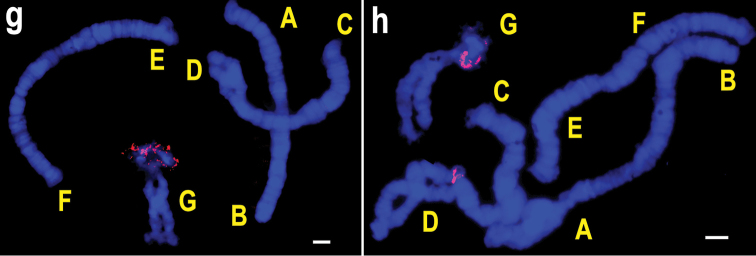
FISH of rDNA probes on polytene chromosomes of species from the subgenus *Chironomus* with multiple localization of hybridization sites. **a** *Chironomus agilis* **b** *Chironomus cingulatus* **c** *Chironomus muratensis* **d** *Chironomus nudiventris* **e** *Chironomus pseudothummi* **f** Chironomus “annularius” **g** *Chironomus balatonicus* with one NOR **h** *Chironomus balatonicus* with additional NOR in arm D. Letters designate chromosomal arms. Green arrows show sites of weak hybridizations signals. Bar = 10 µm.

**Figure 4.**
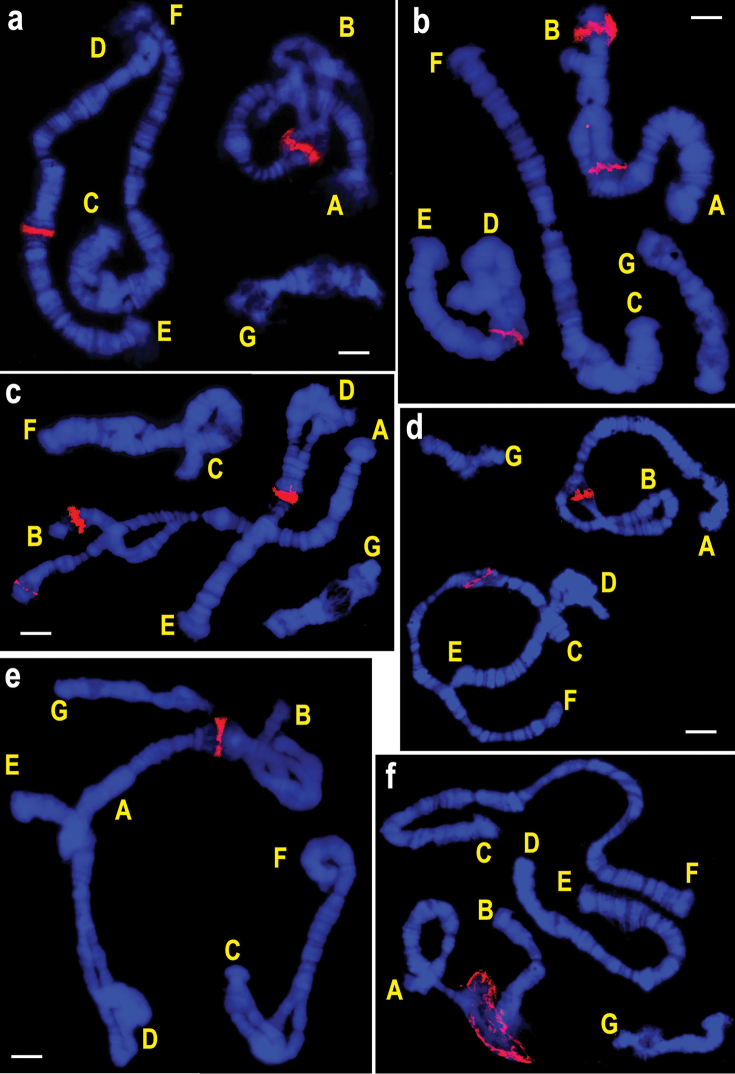
FISH of rDNA probes on polytene chromosomes of species from the subgenus *Camptochironomus*. **a–c** *Chironomus tentans*, where **b** and **c** specimens with heterozygous inversions in arm B that change the position of NOR in one of the homologues **d** *Camptochironomus dilutus*; **e** *Camptochironomus pallidivittatus* **f** *Camptochironomus setivalva*. Letters designate chromosomal arms. Bar = 10 µm.

Karyotypes of most *Chironomus* species studied in this work have four polytene chromosomes, which corresponds to the haploid chromosome set n = 4 (Figs 1–4). The only exception is *Chironomus nudiventris* that have three chromosomes in its haploid set. The chromosome number in the karyotype of this species reduced via fusion of arms G (chromosome IV) and E (chromosome III, EF) to form the joint chromosome GEF (Fig. 3d, Table 3).

DNA-probe used for FISH analysis consists of two main components: gene coding 5,8S rRNA and internal transcribed spacer (ITS1). The percent of identity between ITS-1 sequences of the *Chironomus* species used for producing DNA probes is considerably lower as compared with the percent of identity between their 5.8S rDNA sequences (Table 2). However, FISH has demonstrated that despite considerable interspecific differences in ITS-1, if the probe contains conserved 5.8S rDNA sequences, the number and location of hybridization sites for homologous and heterologous marker DNAs in the karyotypes of examined chironomid species completely coincide (Fig. 1). It should be also noted that the hybridization sites of rDNA probes in most of the studied species coincide with the positions of NORs on chromosomes. This suggests that hybridization of the DNA probes to the chromosomes is mainly determined by the 5.8S rDNA nucleotide sequences and that the regions where rDNA probes hybridize to chromosomes are NORs.

**Table 2.** The percent of identity between 5.8S rDNA nucleotide sequences (above) and ITS-1 (below) in *Chironomus* species.

	*Chironomus agilis*	*Chironomus balatonicus*	*Chironomus muratensis*	*Chironomus plumosus*	Chironomus “annularius”	*Chironomus riparius*	*Chironomus dorsalis*	*Camptochironomus dilutus*	*Camptochironomus pallidivittatus*	*Chironomus tentans*
*Chironomus agilis*		**100**	**100**	**100**	**99**	**99**	**100**	**100**	**100**	**100**
*Chironomus balatonicus*	92		**100**	**100**	**99**	**99**	**100**	**100**	**100**	**100**
*Chironomus muratensis*	94	95		**100**	**99**	**99**	**100**	**100**	**100**	**100**
*Chironomus plumosus*	91	94	96		**99**	**99**	**100**	**100**	**100**	**100**
Chironomus “annularius”	87	90	87	87		**98**	**100**	**100**	**100**	**100**
*Chironomus riparius*	75	76	76	77	76		**100**	**100**	**100**	**100**
*Chironomus dorsalis*	74	78	76	76	73	80		**100**	**100**	**100**
*Camptochironomus dilutus*	76	79	77	78	79	73	73		**100**	**100**
*Camptochironomus pallidivittatus*	77	79	78	78	79	73	73	96		**100**
*Chironomus tentans*	77	80	79	78	78	74	73	95	98	

The number of NORs in the studied *Chironomus* species is different (Figs 1–4, Table 3). Only one chromosome site of rDNA probe hybridization is observed in 12 chironomid species; two sites, in five species; and three, five, or six sites, in the remaining four species (Table 3).

**Table 3.** The number of chromosome pairs in karyotype, combinations of arms in chromosomes, and number and locations of nucleolus organizer regions (NORs) in *Chironomus* species.

Species	Number of chromosome pairs in karyotype	Arm combination in chromosomes	Number of NORs	NOR location	
				Chromosome arm	Chromosome region
Subgenus *Chironomus*					
1. *Chironomus agilis*	4	AB CD EF G	2	G	1a, 1bc^†^
2. *Chironomus balatonicus*	4	–//–	2	G D	1^†^ 18fg^‡^
3. *Chironomus borokensis*	4	–//–	1	G	1^†^
4. *Chironomus entis*	4	–//–	1	G	1^†^
5. *Chironomus muratensis*	4	–//–	6	B C D F G	24i-j^†^ 16^‡^ 2h, 2d^‡^ 10c^‡^ 1^†^
6. *Chironomus plumosus*	4	–//–	1	G	1^†^
7. *Chironomus nudiventris*	3	AB CD GEF	3	G D	1^†^ 2h, 2d^‡^
8. Chironomus “annularius”	4	AB CD EF G	5	A C E G	3g^‡^ 15c–17b^‡^ 3a–4h, 9–10b^‡^ not mapped
9. *Chironomus riparius*	4	–//–	1	G	3^§^
10. *Chironomus cingulatus*	4	–//–	2	B G	not mapped not mapped
11. *Chironomus nuditarsis*	4	–//–	1	G	not mapped
12. *Chironomus sororius*	4	–//–	1	G	not mapped
13. *Chironomus lacunarius*	4	AD BC EF G	1	G	not mapped
14. *Chironomus dorsalis*	4	AE BF CD G	1	G	not mapped
15. *Chironomus luridus*	4	–//–	1	G	not mapped
16. *Chironomus melanescens*	4	–//–	1	G	not mapped
17. *Chironomus pseudothummi*	4	–//–	3	G C G	not mapped 4d^‡^ not mapped
Subgenus *Camptochironomus*					
18. *Camptochironomus dilutus*	4	AB CF ED G	2	B C	9^|^ 10^|^
19. *Camptochironomus pallidivittatus*	4	–//–	1	A	12^|^
20. *Camptochironomus setivalva*	4	–//–	1	B	9^|^
21. *Chironomus tentans*	4	–//–	2	B D	9a–b^|^ 9b^|^

† mapping according to Maximova-Shobanov system (Maximova 1976, Shobanov 1994)

‡ mapping according to Keyl-Devai system (Keyl 1962, Devai et al. 1989)

§ mapping according to Keyl-Hagele system (Keyl 1957, Hagele 1970

| mapping according to Beermann system (Beermann 1955)

The species belonging to the subgenera *Chironomus* and *Camptochironomus* are similar in the number of rDNA loci in their karyotypes (one or two NORs) but differ considerably in their chromosomal positions. In species from the subgenus *Chironomus* NORs have been found in all chromosomal arms, whereas in species from the subgenus *Camptochironomus* NORs have been detected in arms A, B, C and D only. Unlike species belonging to the subgenus *Chironomus* with obligatory presence of one of the NORs in arm G (Figs 2–3), no NOR in this arm has been detected in all four *Camptochironomus* species (Fig. 4). In *Camptochironomus* NOR is most frequently found in arm B, being observed in three of the four examined species, namely, *Camptochironomus setivalva*, *Camptochironomus dilutus*, and *Chironomus tentans*. In the *Camptochironomus setivalva* karyotype it is the only one NOR found, while *Camptochironomus dilutus* and *Chironomus tentans* carried one additional NOR in arms C and D, respectively. Only one NOR has been found in arm A of *Camptochironomus pallidivittatus* (Fig. 4).

Seven species from the subgenus *Chironomus* carried rDNA hybridization sites in other chromosomal arms besides the NOR in arm G. These species can be divided into three groups according to the hybridization pattern of DNA probes.

Two NORs are always observed in the karyotypes of the first group (*Chironomus agilis* and *Chironomus cingulatus*) and the hybridization sites of DNA probes are similar in the intensity of hybridization and completely coincide with the localized NORs. Both NORs of *Chironomus agilis* are located in arm G (one in the centromeric and the other in the telomeric regions); as for the *Chironomus cingulatus* NORs, they are located on arms B and G (Fig. 3a, b, Table 3).

The second group includes species with the number of hybridization sites for DNA probes exceeding the number of cytologically identifiable NORs and with the intensity of hybridization varying between hybridization sites (*Chironomus muratensis*, *Chironomus nudiventris*, and *Chironomus pseudothummi*). In the karyotype of *Chironomus muratensis*, two strong rDNA hybridization signals are always detected in regions developing NORs – one in arm G and the other in arm C, and in addition, weak hybridization signals varying in their intensity and number are detected in arms B, C, D, and F in the regions, where a developed nucleolus has never been observed (Fig. 3c, Table 3). Three rDNA hybridization signals are detected in the karyotypes of *Chironomus nudiventris* and *Chironomus pseudothummi*; the strongest signal is located in arm G and coincides with the active NOR in both species, while two weaker signals are located in arm D region 2h–d in *Chironomus nudiventris* and arms G and C in *Chironomus pseudothummi* (Fig. 3d, e, Table 3). It is necessary to note that active NORs were never been detected in regions with weak hybridization signals in karyotypes of these three species.

The number of NORs in the karyotypes of the third group of species (*Chironomus balatonicus* and Chironomus “annularius”) may vary, however the hybridization sites of DNA probes always coincide with the active NORs (Fig. 3f–h, Table 3). The karyotype of *Chironomus balatonicus* may have one or two NORs: one constantly present in arm G and the other, polymorphic, in arm D (Fig. 3g, h). However, rDNA hybridization signals were detected in arm D only if the arm carried one of the banding sequences balD3, balD17, or balD23. No hybridization signals or developed nucleoli were observed in other arm D banding sequences of *Chironomus balatonicus* (Fig. 3g).

The karyotype of Chironomus “annularius” has either four or five NORs. Four NORs are found in all studied specimens (two NORs in arm E and one in each of arms C, and G). An intraspecific NOR polymorphism has been observed in Chironomus “annularius” arm A, region 3g (Fig. 3f). NOR localized to this region is present in either homozygous or heterozygous state in approximately 70% of the larvae (Kiknadze et al. 2012). The rDNA hybridization signals in arm A have not been detected in one-third of the examined larvae, which coincide with the absence of active NOR in this region.

## Discussion

The genus *Chironomus* comprises over 150 species (Shobanov et al. 1996). The karyotypes of these species are usually studied using the salivary gland polytene chromosomes rather than mitotic or meiotic chromosomes as in the majority of other insect species. The fact is that the mitotic and meiotic chromosomes of chironomids are very tiny, 1–5 µm, which prevents from distinguishing secondary constrictions and other chromosome markers, while karyotypes of species are very similar. Polytene chromosomes of chironomids are considerably longer. The average lengths of *Chironomus riparius* metacentric and submetacentric polytene chromosomes (I–III) are 110, 100, and 85 µm, respectively, and the shortest acrocentric chromosome (IV) reaches 30 µm (Kiknadze and Gruzdev 1970). Four chromosomes of chironomid haploid karyotype comprise over 1000 precisely mapped bands (Kiknadze et al. 2004a, b). Thus, the density of markers (bands and interbands) is sufficient to robustly identify individual species, detect chromosome rearrangements, and study chromosome evolution of the genus *Chironomus*.

NORs are additional markers in polytene chromosomes. Nucleoli are actively transcribed regions of chromosomes, visible on chromosomes as giant puffs. Phase contrast microscopy of orcein-stained chromosomes allows them to be distinguished from any other functionally active chromosome regions (Beermann 1960). However, the activity of nucleoli significantly varies during chironomid development (Kiknadze et al. 1981, Razmakhnin et al. 1982), creating certain problems with their precise identification. Most of these problems can be resolved by the use of *in situ* hybridization, AgNO_3_ staining, and FISH (Hollenberg 1976, Eigenbrod 1978, Lentzios and Stocker 1979, Razmakhnin et al. 1982). The data on NORs detected using silver staining in 11 Australian chironomid species (Lentzios and Stocker 1979) and FISH in 21 Palearctic chironomid species (this work) demonstrate that the number and locations of the NORs detected in polytene chromosomes of the examined species mainly coincide with their number and locations determined by phase contrast microscopy, although there were several exceptions from these rule.

In some cases the number of NORs detected by FISH or AgNO_3_ staining does not match to number of NORs detected by phase contrast analysis. In particular, staining with AgNO_3_ detected six NORs in the polytene chromosomes of *Chironomus duplex* Walker, 1856 salivary gland, but only one NOR in interphase ganglion cells and none in the meiotic late prophase and metaphase I as well as mitotic chromosomes. The authors assume that the observed differences are determined by tissue-specific features in the NOR function, namely, fusion of nucleoli in ganglion cells and a decrease in the NOR transcription activity after the pachytene in meiosis (Lentzios and Stocker 1979). This phenomenon is characteristic not only of chironomids, but also of other insect groups (Cabrero and Camacho 2008, Cabral-de-Mello et al. 2010, Grzywacz et al. 2011).

One of the possible reasons underlying the variation in NOR activity in chironomids is a change in the number of transcriptionally active copies of ribosomal genes. A special study into the chromatin structure of *Chironomus riparius* ribosomal genes has shown that not all these copies are equally active in transcribing rRNA. Along with transcriptionally active copies of ribosomal genes, free of nucleosomes, populations of these genes also contain transcriptionally inactive copies displaying nucleosome organization. The share of transcriptionally active copies in the population of ribosomal genes is tissue-specific, amounting to 80% in the fat body cells, to 50% in the salivary glands, and only 20% in the Malpighian tube cells (Sanz et al. 2007). An analogous ratio is observed in the *Chironomus tentans* salivary gland cells, where 40% of the ribosomal genes are in a transcriptionally active state (Madalena et al. 2012). Since silver staining predominantly detects active NORs, the variation in NOR number observed in chironomids using this technique may be actually determined by the variation in the transcriptional activity of their ribosomal genes. However, this factor does not influence the NOR detection by FISH.

Variability in activity of NORs might be also determined by such characteristics of this locus as multiple copies of rRNA genes and a presence of transposable elements (TE) (Long and Dawid 1980, Jakubczak et al. 1991). The presence of multiple gene copies allows part of them to be separated by crossing-over, while mobile elements enhance their transfer to new genomic regions. If these events do not involve regulatory sites for ribosomal genes, then localization and activity of the initial NOR are retained and additional new NORs appear; the activity of the latter depends on the rDNA copy number in the transferred fragment (Eickbush and Eickbush 2007). The number of ribosomal gene copies in these fragments may be different, as demonstrated by the length variation of extrachromosomal circular DNA (eccDNA) formed by multimers of tandemly repeated rDNA genes (Cohen et al. 2003, 2010) as a result of recombination between adjacent gene clasters and intergene spacers.

If this mechanism is involved, the variability in intensity of hybridization signals of rDNA on chromosomes of *Chironomus muratensis*, *Chironomus nudiventris* and *Chironomus pseudothummi* might be determined by the difference in the number of gene copies presented in each NOR. Thus, intense hybridization signals were detected in regions with active NORs while weak signals occurred in regions with no visible NOR activity. A similar pattern has been observed in wheat (Dubcovsky and Dvořák 1995) and many other species (Cabrero and Camacho 2008).

The analysis involving FISH and silver staining has shown a considerable diversity in the NOR number and locations in the chromosomes constituting karyotypes of 32 Palearctic and Australian *Chironomus* species (Lentzios and Stocker 1979; our data, Table 3). The number of NORs in these species may vary from one to eight. The variant with a single NOR is prevalent in the chironomid karyotypes, being observed in 17 of the 32 examined species; two NORs are found in eight species; and three NORs in three species. Four species contain considerably larger number of NORs, namely, five (Chironomus “annularius”), six (*Chironomus duplex* and *Chironomus muratensis*), and eight (*Chironomus nepeanensis*).

NORs can be located on all seven chromosome arms of the chironomid karyotypes; however, none of the NORs have been detected on the same chromosome arm in all 32 species of the genus *Chironomus*. Most frequently, NOR is located on arm G (in 25 species out of 32), but none of the species belonging to the subgenus *Camptochironomus* had NOR on this arm. The locations of NORs is also different in species from the subgenus *Chironomus* inhabiting remote geographic regions: species from Western Siberia may carry NOR in all chromosome arms (Table 3), while the Australian species lack NOR in arm E (Lentzios and Stocker 1979). The interspecific differences in the NOR number and location are also observed in closely related chironomid species.

Along with the interspecific variation in the NOR number and location, chironomids also display intraspecific variation in these characteristics. Three species (*Chironomus balatonicus*, Chironomus “annularius”, and *Chironomus tepperi*) may carry different numbers of NORs in individual karyotypes. *In situ* hybridization of *Chironomus tepperi* chromosomes with 28S rRNA (Eigenbrod 1978) and FISH of *Chironomus balatonicus* and Chironomus “annularius” chromosomes with 5.8S rDNA (this work) have demonstrated that the additional NORs develop only in the individuals that carry ribosomal genes in the corresponding chromosome regions.

Variety in number and locations of NOR on chromosomes in karyotypes of species from the genus *Chironomus* can occur due to several reasons: as a result of chromosomal rearrangemens, mainly inversions and translocations that are widespread in chironomids (Kiknadze et al. 2008), in consequence of transpositions of chromosomal fragments containing NORs into other regions of homologous and non-homologous chromosomes due to sister chromatid exchanges, homologous recombination, crossing-over or other mechanisms. All of this can result in an emergence of NORs in regions where they did not occur before or in a loss of NORs from regions of their traditional occurrence.

Transposable elements (TE) can also cause considerable changes in organization of NORs in karyotypes of species from the genus *Chironomus*. They can change activity of NORs or cause their complete inactivation. Several types of TE were found in the genus *Chironomus*. Common features for all of them are the presence in genome of multiple copies of each element, multiple location sites, species-specific but demonstrate intraspecific, intra- and interpopulations variability (Papusheva et al. 2004, Zampicinini et al. 2011). All of this allow to consider TE as a source that can provide a possibility for transpositions and changes in number of NORs on chromosomes of different species of the genus *Chironomus*.

**Figure 5.**
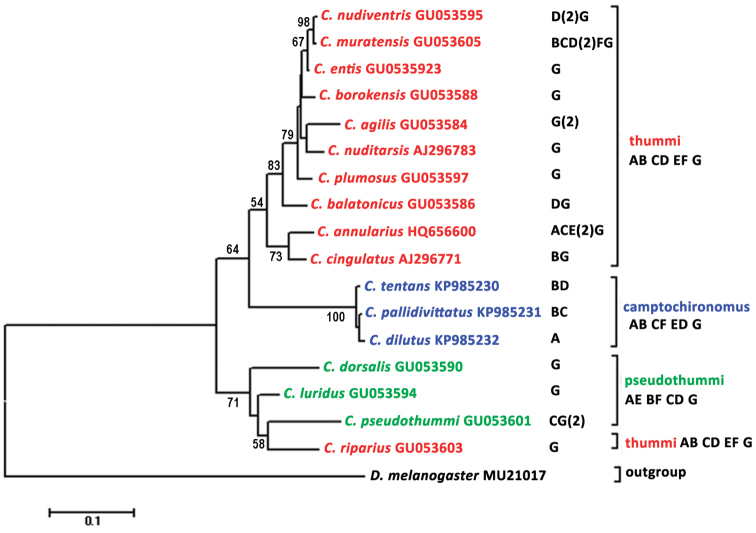
NJ tree based on maximum likelihood distances for ITS1 and 5,8S rDNA sequences from the genus *Chironomus* species. *Drosophila melanogaster* is used as an outgroup species. Maximum likelihood bootstrap values (1000 replicates) (> 50%) are shown next to the nodes. NORs chromosomal arms location, arm combinations and name of cytocomplexes are listed at the right.

The obtained results allowed us to characterize chromosomal organization and evolution of rRNA genes family in the genus *Chironomus*. The tree of phylogenetic relationships between species from the genus *Chironomus* constructed on the basis of comparison sequences of ITS1 and 5,8S rDNA shows that species groups into tree distinct clusters that coincide with cytocomplexes that differ from each other by arm combinations in chromosomes (Fig. 5). The phylogenetic tree demonstrates mostly monophyletic evolution of rRNA genes in these species. The only exception is *Chironomus riparius*, which belong to “thummi” cytocomplex on the basis of chromosomal arm combination but is clustered in the “pseudothummi” cytocomplex on the tree. It should be noted that the same picture can be observed on phylogenetic trees constructed on the basis of other markers, such as isozymes (Scholl et al. 1980), genes from nuclear and mitochondrial genomes (Guryev et al. 2001) or banding sequences (Shobanov 2002, Kiknadze et al. 2004b, 2008, Gunderina et al. 2005). According to hypothesis of Keyl (1962) the reason for such behavior is that originally *Chironomus riparius* belonged to the “pseudothummi” cytocomplex but undergone the change in chromosome arm combination due to reciprocal translocation between chromosomes AE and BF, which resulted in its transfer into “thummi” cytocomplex. But if such an event occurred relatively recently in this species evolution its genome has not accumulate enough changes to differ it from other species from the “pseudothummi” cytocomplex.

Addition of data on the number and chromosomal positions of NORs to the phylogenetic tree of studied chironomid species shows that there is no correlation between evolution of nucleotide sequences of ribosomal genes and chromosomal organization of NORs in the karyotypes of species (Fig. 5). The analysis had shown that number and location of NORs in karyotypes had changed many times during evolution of the genus *Chironomus* while evolution of ribosomal genes was monophyletic.

At the same time the combined data allow us to suggest a hypothesis about location of NOR in the karyotype of an ancestor species of the genus *Chironomus*. As all species from both “thummi” and “pseudothummi” cytocomplexes always have one NOR in arm G it is possible to suppose that an ancestor chironomid species had a NOR in this arm. And the absence of NOR in the arm G of species from the “camptochironomus” cytocomplex is probably caused by its loss in the ancestor species of this cytocomplex after its separation from “thummi” cytocomplex.

## References

[B1] Beermann W (1960) Der Nukleolus als lebenswichtiger Bestandteil des Zellkernes. Chromosoma 11: 263–296. doi: 10.1007/BF00328655 13798246 10.1007/BF00328655

[B2] Cabrero J Camacho JPM (2008) Location and expression of ribosomal RNA genes in grasshoppers: Abundance of silent and cryptic loci. Chromosome Research 16: 595–607. doi: 10.1007/s10577-008-1214-x 18431681 10.1007/s10577-008-1214-x

[B3] Cabral-de-Mello DC Moura RC Martins C (2010) Chromosomal mapping of repetitive DNAs in the beetle *Dichotomius geminatus* provides the first evidence for an association of 5S rRNA and histone H3 genes in insects, and repetitive DNA similarity between the B chromosome and A complement. Heredity 104: 393–400. doi: 10.1038/hdy.2009.126 19756039 10.1038/hdy.2009.126

[B4] Cabral-de-Mello DC Oliveira SG Moura RC Martins C (2011) Chromosomal organization of the 18S and 5S rRNAs and histone H3 genes in Scarabaeinae coleopterans: insights into the evolutionary dynamics of multigene families and heterochromatin. BMC Genetics 12: . doi: 10.1186/1471-2156-12-88 10.1186/1471-2156-12-88 PMC3209441 21999519

[B5] Cohen S Yacobi K Segal D (2003) Extrachromosomal circular DNA of tandemly repeated genomic sequences in *Drosophila*. Genome Research 13: 1133–1145. doi: 10.1101/gr.907603 12799349 10.1101/gr.907603 PMC403641

[B6] Cohen S Agmon N Sobol O Segal D (2010) Extrachromosomal circles of satellite repeats and 5S ribosomal DNA in human cells. Mobile DNA 1: . doi: 10.1186/1759-8753-1-11 10.1186/1759-8753-1-11 PMC3225859 20226008

[B7] Devai G Miskolczi M Wülker W (1989) Standartization of chromosome arms B, C and D in *Chironomus* (Diptera, Chironomidae). Advances in chironomidology. Part I. Acta biologica Debrecina: supplementum Oecologica Hungarica 2(1): 79–92.

[B8] Dubcovsky J Dvořák J (1995) Ribosomal RNA multigene loci: Nomads of the Triticeae genomes. Genetics 140: 1367‒1377. 7498776 10.1093/genetics/140.4.1367 PMC1206700

[B9] Edgar RC (2004) MUSCLE: multiple sequence alignment with high accuracy and high throughput. Nucleic Acids Research 32(5): 1792–1797. doi: 10.1093/nar/gkh340 15034147 10.1093/nar/gkh340 PMC390337

[B10] Eigenbrod J (1978) Differences in the number of nucleolus organizers in *Chironomus tepperi* shown by in situ hybridization. Chromosoma 67: 63–66. doi: 10.1007/BF00285648 688844 10.1007/BF00285648

[B11] Eickbush TH Eickbush DG (2007) Finely orchestrated movements: Evolution of the ribosomal RNA genes. Genetics 175: 477–485. doi: 10.1534/genetics.107.071399 17322354 10.1534/genetics.107.071399 PMC1800602

[B12] Golygina VV Istomina AG Rakisheva AZh Kiknadze II (1996) New banding sequences in the *Chironomus balatonicus* karyofund. Tsitologiya 38(8): 869–883. [In Russian]

[B13] Grzywacz B Maryańska-Nadachowska A Chobanov DP Karamysheva T Warchałovska-Śliwa E (2011) Comparative analysis of the location of rDNA in the Palaearctic bushcricket genus *Isophya* (Orthoptera: Tettigoniidae: Phaneropterinae). European Journal of Entomology 108(4): 509–517. doi: 10.14411/eje.2011.066

[B14] Gunderina LI Katokhin AV (2011) Variation and divergence of the rDNA ITS-1 region in species of the genus *Chironomus* (Diptera: Chironomidae) Contemporary Chironomid Studies. Proceedings of the XVII International Symposium on Chironomidae, July 6–9, 2009, Nankai University, Nankai University Press, China, 22–35.

[B15] Gunderina LI (2014) Design of molecular markers for identification of species of the genus *Chironomus* (Diptera, Chironomidae). Entomological Review 94: 140–148. doi: 10.1134/S0013873814010151

[B16] Guryev V Makarevitch I Blinov A Martin J (2001) Phylogeny of the genus *Chironomus* (Diptera) inferred from DNA sequences of mitochondrial *Cytochrome b* and *Cytochrome oxidase I*. Molecular Phylogenetics and Evolution 19: 9–21. doi: 10.1006/mpev.2001.0898 11286487 10.1006/mpev.2001.0898

[B17] Hägele K (1970) DNS-Replikationmuster der Speicheldrüsen-Chromosomen von Chironomiden. Chromosoma 31: 91–138. doi: 10.1007/BF00321159 5489359 10.1007/BF00321159

[B18] Hollenberg CP (1976) Proportionate representation of rDNA and Balbiani Ring DNA in polytene chromosomes of *Chironomus tentans*. Chromosoma 57: 185–197. doi: 10.1007/BF00292917 954553 10.1007/BF00292917

[B19] Jakubczak JL Burke WD Eickbush TH (1991) Retrotransposable elements *R1* and *R2* interrupt the rRNA genes of most insects. Proceedings of the National Academy of Sciences of the United States of America 88: 3295–3299. doi: 10.1073/pnas.88.8.3295 1849649 10.1073/pnas.88.8.3295 PMC51433

[B20] Keyl H-G (1957) Untersuchungen am Karyotypus von *Chironomus thummi*. I. Mitteilung. Karte der Speicheldrüsen-Chromosomen von *Chironomus thummi* und die cytologishe Differenzierung der Subspezies *Ch. th. thummi* und *Ch. th. piger*. Chromosoma 8: 739–756. doi: 10.1007/BF01259532 13523751

[B21] Keyl H-G (1962) Chromosomenevolution bei Chironomus. II. Chromosomenumbauten und phylogenetische Beziehungen der Arten. Chromosoma 13: 464–514. doi: 10.1007/BF00327342

[B22] Kiknadze II Gruzdev AD (1970) Change in chromosome length related to polyteny in the chironomid salivary glands. Tsitologiya 12: 953–960. [In Russian]

[B23] Kiknadze II Panova TM Zakharenko LP (1981) The comparative characteristics of puffing pattern in salivary gland chromosomes during larval development and metamorphosis. III. Transcriptional activity of the nucleolus and the Balbiani Rings. Tsitologiya 23: 531–538. [In Russian]

[B24] Kiknadze II Shilova AI Kerkis IE Shobanov NA Zelentsov NI Grebenjuk LP Istomina AG Prasolov VA (1991) Karyotypes and larval morphology in the tribe Chironomini: an atlas. Nauka, Novosibirsk, 115 pp [In Russian]

[B25] Kiknadze II Golygina VV Istomina AG Gunderina LI (2004a) Pattern of chromosomal polymorphism during population and species divergence in *Chironomus* (Diptera, Chironomidae). Sibirskiy Ekologicheskiy Zhurnal 5: 635–651. [In Russian]

[B26] Kiknadze II Gunderina LI Istomina AG Gusev VD Miroshnichenko LA (2004b) Reconstruction of chromosomal evolution in genus Chironomus. Evrasiatskiy Entomologicheskiy Zhurnal 3: 265–273. [In Russian]

[B27] Kiknadze II Gunderina LI Butler MG Wuelker WF Martin J (2008) Chromosomes and continents. In: Dobretsov N Rozanov A Kolchanov N Zavarzin A (Eds) Biosphere Origin and Evolution. Springer, 349–369. doi: 10.1007/978-0-387-68656-1_25

[B28] Kiknadze II Istomina AG Golygina VV (2012) The karyotype and chromosome polymorphism of the Holarctic species Chironomus «annularius» sensu Strenzke, 1959 (Diptera, Chironomidae). Evrasiatskiy Entomologicheskiy Zhurnal 11: 95–114. [In Russian]

[B29] Lentzios G Stocker AJ (1979) Nucleolar relationships in some Australian *Chironomus* species. Chromosoma 75: 235–238. doi: 10.1007/BF00292210 533672 10.1007/BF00292210

[B30] Long EO Dawid IB (1980) Repeated genes in eukaryotes. Annu. Rev. Biochem. 49: 727–764. doi: 10.1146/annurev.bi.49.070180.003455 6996571 10.1146/annurev.bi.49.070180.003455

[B31] Madalena CRG Diez JL Gorab E (2012) Chromatin structure of ribosomal RNA genes in dipterans and its relationship to the location of nucleolar organizers. PLoS ONE 7(8): . doi: 10.1371/journal.pone.0044006 10.1371/journal.pone.0044006 PMC3431366 22952852

[B32] Martin J (1979) Chromosome as tools in taxonomy and phylogeny of Chironomidae (Diptera). Entomologica Scandinavica 10: 67–74.

[B33] Maximova FL (1976) The karyotype of *Chironomus plumosus* from the Ust’ Izhora wild population of Leningrad region. Tsitologiya 18: 1264–1269. [In Russian].

[B34] Neto MSR deSouza MJ Loreto V (2013) Chromosomal evolution of rDNA and H3 histone genes in representative Romaleidae grasshoppers from northeast Brasil. Molecular Cytogenetics 6: . doi: 10.1186/1755-8166-6-41 10.1186/1755-8166-6-41 PMC3853473 24090216

[B35] Oliveira NL Cabral-de-Mello DC Rocha MF Loreto V Martins C Moura RC (2011) Chromosomal mapping of rDNA and H3 histone sequences in the grasshopper *Rhammatocerus brasiliensis* (Acrididae, Gomphocerinae): extensive chromosomal dispersion and co-localization of 5S rDNA/H3 histone clusters in the A complement and B chromosome. Molecular Cytogenetics 4: . doi: 10.1186/1755-8166-4-24 10.1186/1755-8166-4-24 PMC3234176 22075079

[B36] Papusheva E Grul MC Berezikov E Groudieva T Scherbik SV Martin J Blinov A Bergtrom G (2004) The evolution of SINEs and LINEs in the genus *Chironomus* (Diptera). Journal of Molecular Evolution 58: 269–279. doi: 10.1007/s00239-003-2549-8 15045482 10.1007/s00239-003-2549-8

[B37] Razmakhnin EP Kiknadze II Panova TM Mertvetsov NP Ammosov AD Sidorov BN (1982) The in situ hybridization study of the nucleolus organizer functional activity in polytene chromosomes from different tissues of *Chironomus thummi*. Tsitologiya 24: 863–868. [In Russian]

[B38] Sanz C Gorab E Ruiz MF Sogo JM Diez JL (2007) Chromosomal structure of ribosomal genes in *Chironomus thummi* (Diptera: Chironomidae): tissue specificity and behaviour under drug treatment. Chromosome Research 15(4): 429–438. doi: 10.1007/s10577-007-1134-1 17487564 10.1007/s10577-007-1134-1

[B39] Scholl A Geiger HJ Ryser HM (1980) Die Evolution der Gattung *Chironomus* aus Biochemisch-Genetischer Sicht. In: Murray DA (Ed.) Chironomidae. Ecology, Systematics, Cytology and Physiology, Oxford, New York, 25–33. doi: 10.1016/B978-0-08-025889-8.50009-X

[B40] Shobanov NA (1994) The karyofund of *Chironomus plumosus* (L.) (Diptera, Chironomidae). II. Banding pattern of chromosome arms. Tsitologiya 36: 123–128. [In Russian]

[B41] Shobanov NA (2002) Evolution of the genus *Chironomus* (Diptera, Chironomidae). 2. Phylogenetic model. Entomological Review 82: 584–592.

[B42] Shobanov NA Shilova AI Belyanina SI (1996) The composition and structure of the genus *Chironomus* Meigen (Diptera, Chironomidae): a review of the world fauna. In: Shobanov NA Zinchenko TD (Eds) Ecology, Evolution and Systematics of Chironomid Midges. Inst. Biol. of Inland Waters, Togliatti, 44–96. [In Russian]

[B43] Tamura K Stecher G Peterson D Filipski A Kumar S (2013) MEGA6: Molecular Evolutionary Genetics Analysis version 6.0. Molecular Biology and Evolution 30(12): 2725–2729. doi: 10.1093/molbev/mst197 24132122 10.1093/molbev/mst197 PMC3840312

[B44] Wuelker W (1980) Basic patterns in the chromosome evolution of the genus *Chironomus* (Diptera). Zeitschrift für zoologische Systematik und Evolutionsforschungen 18: 112–123. doi: 10.1111/j.1439-0469.1980.tb00733.x

[B45] Zampicinini G Cervella P Biémont C Sella G (2011) Insertional variability of four transposable elements and population structure of the midge *Chironomus riparius* (Diptera). Molecular Genetics and Genomics 286: 293–305. doi: 10.1007/s00438-011-0646-8 21901555 10.1007/s00438-011-0646-8

